# What Does It Take to Be an Effective National Steward of Digital Health Integration for Health Systems Strengthening in Low- and Middle-Income Countries?

**DOI:** 10.9745/GHSP-D-18-00270

**Published:** 2018-10-10

**Authors:** Michael J. Frost, Jacqueline B. Tran, Fatema Khatun, Ingrid K. Friberg, Daniela C. Rodríguez

**Affiliations:** a Norwegian Institute of Public Health, Oslo, Norway.; b Health Information Systems Programme, Department of Informatics, University of Oslo, Oslo, Norway.; c Department of International Health, Johns Hopkins Bloomberg School of Public Health, Baltimore, MD, USA.; d International Centre for Diarrhoeal Disease Research, Bangladesh, Dhaka, Bangladesh.

## Abstract

**Background:**

Digital innovations have evolved over the last 15 years to support health activities, and their introduction in low- and middle-income countries has shown the potential to catalyze gains in health systems and service delivery. Despite widespread efforts to roll out these technologies, standardized approaches for formalizing national stewardship responsibilities and ensuring that digital health is a routine, mature, sustainable, and country-owned component of the health system are lacking. In this paper, we define digital health stewardship, with a focus on the ministry of health's role; describe practices undertaken to date; and identify gaps where increased attention could improve sustainability, impact, and local ownership.

**Methods:**

We conducted a purposeful review of peer-reviewed and gray literature. Of the 404 identified resources from the peer-reviewed literature, 12 met all of the inclusion criteria. After searching various online gray literature repositories, we identified 6 sources based on their quality, source, and relevance. Selected resources were abstracted for relevance to our stewardship themes and synthesized.

**Results:**

Findings are presented in 4 broad thematic areas: strategic direction, policies and procedures, roles and responsibilities, and health service delivery implications. Evidence related to strategic direction offers guidance on the main responsibilities under digital health stewardship, including regulations and incentives to promote compliance with standards, mechanisms for oversight, and structures to support evidence-based decisions, and the potential institutional structures and goals that could be used to achieve them. A number of examples of high-level policies and implementation-oriented procedures, such as from the European Commission and the World Health Organization, demonstrate how to operationalize the strategic direction. Available evidence for the remaining themes was sparse, drawing attention to key areas for future work.

**Conclusions:**

Despite the importance of country-owned stewardship of digital health, the guidance available is limited and aspirational. Concrete recommendations, including how to adapt existing innovations to the local context, are needed. In particular, the role of external partners needs to be oriented toward building and supporting country capacity to achieve digital health stewardship's potential to support health systems into the future.

## Abstract (precis)

A purposeful literature review of peer-reviewed and gray literature identified 4 broad thematic areas of digital health stewardship—strategic direction, policies and procedures, roles and responsibilities, and health service delivery—that need further research and development in order for digital health to be better positioned to positively impact low- and middle-income country health systems.

## INTRODUCTION

In recent years, the use of digital technologies to support global public health efforts has become common, with donors, nongovernmental organizations (NGOs), technology providers, and ministries of health (MOHs) all providing varying levels of attention and support to a range of digital approaches. Public health leaders such as the World Health Organization (WHO), the U.S. Centers for Disease Control and Prevention the United States Agency for International Development, and the Global Fund to Fight AIDS, Tuberculosis and Malaria have written about, funded/invested in and implemented a wide variety of digital health approaches and interventions, signaling a general consensus about digital health's importance for public health now and in the future.

The term ‘digital health’ encompasses eHealth, information and communication technology (ICT) for public health^1^; mHealth, mobile wireless technologies for public health^2^; as well as novel areas of technology with roots in computing but applied to health. The most common interventions within this increasingly broad area of activity can be categorized into 3 overlapping domains: (1) support for health services and outcomes, such as point-of-care diagnostics and behavior change communication messaging to patients; (2) provider-targeted tools for clinical decision support, work planning, training, and management; and (3) digitization of routine health systems functions such as electronic health records, vital event registries, supply chain management, and financial transactions.^3^

In May 2018, the World Health Assembly adopted a digital health resolution urging member states to assess and consider how to appropriately include digital technologies in their health systems.^4^ Documentation highlighted such issues as multiplicity of pilot projects, lack of interconnectedness, absence of standards and tools, and lack of multisectoral approaches within government and agencies.^2^ This resolution clearly demonstrates that proper stewardship of this complex digital health landscape by MOHs is crucial to ensure impact and sustained success beyond the life of externally funded and/or driven initiatives. A shift from the current environment of mostly project-led, externally funded, siloed interventions to sustainable, integrated, locally owned and managed digital health programs is needed. Without changing the landscape to systematically support and guide country leadership, the field of digital health will likely continue to be characterized by small one-off projects and short-lived activities.^5^ Unfortunately, guidance to accomplish these goals has been sorely lacking.^6^ Improper stewardship can result in not only wasted resources on low-impact digital health activities and the diversion of resources from other more proven health interventions but also missed opportunities for promising technological breakthroughs and evidence-based strategies to improve efficiencies within routine systems. Digital health interventions can only fully realize their promise if they become routine, locally managed, properly stewarded approaches, but what would this entail?

Digital health interventions can only fully realize their promise if they become routine, locally managed, properly stewarded approaches.

In this paper, we focus on defining stewardship for digital health—and identifying current trends and gaps to address in the future—as a foundational topic in support of the other papers in this issue. For more complete discussions related to digital health's contribution to health workforce, service receipt, demand generation, service provision/delivery, and financing, see the rest of the papers in this series.

### Stewardship of Digital Health

The goal of a locally managed digital health environment implies proper stewardship and governance of the entire system. For the purposes of this paper, we are using ‘stewardship’ as a broader term than ‘governance.’ Our definitions build upon a comparison of the International Organization of Standardization (ISO) standards 38500:2015^7^ and 14639-2:2014^8^ on governance of information technology (IT) and health informatics, respectively; the Broadband Commission for Sustainable Development's report on digital health;^5^ and the WHO definition for health systems stewardship.^11^ We further clarify that stewardship for digital health includes considerations beyond those relevant for health systems and offer a complete definition.

Under ISO, governance of IT “provide[s] principles, definitions, and a model for governing bodies to use when evaluating, directing, and monitoring the use of information technology […] in their organizations.”^7^ This standard provides guidance with regard to 6 specific principles: (1) responsibility, (2) strategy, (3) acquisition, (4) performance, (5) conformance, and (6) human behavior.^7^ While many other standards-related IT applications for health care exist, they lack a usable overview that combines the health, privacy, security, human resource, evidence, sustainability, and investment considerations that together lead to a comprehensive stewardship approach.

The Broadband Commission for Sustainable Development defines ‘governance’ in terms of mechanisms: “the means by which intragovernmental and cross-sectoral collaboration is organized by entities that advise, coordinate, support, regulate, monitor, and implement digital health services and applications, and ensure the security of the health information exchange.”^5^ They also highlight the function of a governance mechanism to engage stakeholders across the public and private sector.

The WHO definition of health system stewardship contains some of this context, but does not emphasize the specific considerations relevant to the use of digital health technology. The definition states that ‘stewardship’ [emphasis ours]^11^:

refers to the wide range of functions carried out by governments as they **seek to achieve national health policy objectives**. In addition to improving overall levels of population health, **objectives are likely to be framed in terms of equity, coverage, access, quality, and patients' rights**. National policy may also **define the relative roles and responsibilities of the public, private and voluntary sectors – as well as civil society** – in the provision and financing of health care.

*Stewardship is a political process that involves balancing competing influences and demands. It will include: **maintaining the strategic direction of policy development and implementation; detecting and correcting undesirable trends and distortions; articulating the case for health in national development; regulating the behavior of a wide range of actors – from health care financiers to health care providers; and establishing effective accountability mechanisms***. […]

*A key concern in many countries is to build the capacity needed to carry out stewardship functions effectively. This, in turn, requires a better understanding of what constitutes best practice when it comes to stewardship and **how national leadership can be developed.** It is increasingly recognized that the provision of development assistance needs to be geared to fulfilling these objectives.*^9^

This definition highlights not only the overarching function of supporting the attainment of high-level policy objectives but also the more specific components of that function. More succinctly, health system stewardship entails setting a strategic direction, guiding the policy and procedures to achieve that vision, defining and regulating the roles and responsibilities of actors in the system, and ensuring that critical health service delivery implications, like equity and access, are regularly and systematically addressed.

While digital health stewardship shares many aspects with health systems stewardship practices, such as informed policymaking or cost-benefit or effectiveness analyses, there are number of unique tasks that differ as well, such as integrating routine technology-related trainings and education programs for health staff, regular landscape scanning for both potential interventions and activities that would benefit from a digital update, approaches for combining and integrating digital health technologies into an overarching and sustainable ecosystem, and innovative thinking about how to adapt digital health strategies to local facilities and practitioners with limited resources. MOHs operate within the constraints of significant resource challenges, especially in low- and middle-income countries (LMICs)—not only in terms of funding but also in terms of technical and human resource capacity. Decades of investment in education, training, infrastructure, policies, and health commodities has helped make many public health interventions routine in LMICs; however, the investments needed to fully prepare countries to adopt a new set of digital strategies that, in many ways fall outside of the norm of public health activities, are still missing.

We, thus, offer the following definition for digital health stewardship:

**Digital health stewardship** refers to the comprehensive national actions and policies required to ensure the appropriate, sustainable, routine, and safe use of digital health technologies within the broader national health and IT domains. Located at the intersection of health and technology, digital health stewardship necessarily includes considerations from both fields, including clearly established approaches for evaluation and selection of technologies; integration of technologies and health practices into a combined ecosystem; governance of sensitive health information; routinized training, education, and support for health staff; dedicated financial streams for ongoing support; standardized IT-support mechanisms; and policies/legislation governing each of these domains.

Digital health stewardship refers to the comprehensive national actions and policies required to ensure the appropriate, sustainable, routine, and safe use of digital health technologies within the broader national health and IT domains.

In order to gain and maintain an effective stewardship role, MOHs need to provide leadership and capacity to deliver on the abovementioned functions and ensure more locally owned and driven responses. The challenge of local ownership is not solely a digital health domain issue.^10^ Public health, in general, is influenced by a variety of global actors, both public and private, seeking to affect critical health areas irrespective of national borders. To that end, there are many examples of global and national actors interacting over policies—from child health to HIV to tobacco—that highlight the role and power or powerlessness of LMIC MOHs as they attempt to exert their governance role.^11^^–^^13^ For digital health to enter a stage of maturity, improved models for stewardship at the national level must be developed, adopted, and supported, with actors across the digital health space leveraging their contributions without undermining local capacity, ownership, and sustainability.

The Sustainable Development Goals^14^ and the pursuit of universal health coverage,^15^ with their explicit and implicit reliance on digital health technologies, suggest opportunities for countries, especially LMICs, to take on the challenge of digital health stewardship as part of broader efforts to improve efficiency and effectiveness of health systems and have a greater impact on health. In this paper, we review the available evidence and current recommendations concerning best practices for stewardship of digital health innovations and present the current state of knowledge about proper stewardship and explore gaps in that knowledge.

## METHODS

Our goal was to identify lessons that can be drawn from existing literature to help ensure an effective stewardship of digital health projects, and to suggest what practices MOHs, donors, NGOs, and other groups can adopt to increase the likelihood of proper stewardship.

To identify these lessons, from July to September 2017, we conducted a purposeful review of both peer-reviewed and gray literature using expansive search terms to reflect the shifting language and changing contexts around stewardship of digital health technologies. For the peer-reviewed literature, we searched Scopus and Web of Science using a combination of digital health terms with ‘governance’ or ‘stewardship’ to ensure a complete literature review (Table 1). Terms focused primarily on service delivery, such as ‘telemedicine,’ or on data analysis techniques, such as ‘big data,’ were purposively excluded from these searches. For the gray implementation-focused literature, we conducted a search for documents in several repositories and websites, including those belonging to GSMA, an association representing mobile operators worldwide; the U.S. President's Emergency Plan for AIDS Relief; the Vodafone Foundation; the United Nations Foundation's Mobile Alliance for Maternal Action project; and the World Health Organization as well as the Knowledge for Health project's mHealth Evidence (www.mhealthevidence.org) and mHealth Knowledge (www.mhealthknowledge.org) websites and HingX.com, a website serving as repository for health ICT initiatives. We also searched Google and Google Scholar and used the snowballing method to identify additional sources from references cited in various fact sheets, conference proceedings, and book chapters.

**TABLE 1.** Literature Search Strategy

Database Searched	Search Terms	Limits	Initial Search Results	Included in Review
**Peer-Reviewed Literature**				
Scopus and Web of Science	Governance/stewardship/accountability PLUS Digital health/eHealth/electronic health/mHealth/mobile health	No articles before 1995 Medicine/health/nursing or social science/decision science or environment English or Spanish only	304 (Scopus) + 318 (Web of Science) = 404 deduplicated results	12^16^^–^^27^
**Gray Literature**				
HingX.com and the GSMA, PEPFAR, Vodaphone Foundation, MAMA, mHealth Evidence and mHealth Knowledge by K4Health, Google, WHO, and others	Governance/stewardship PLUS eHealth/mHealth/digital health/mobile health/individual data/subject tracker/registry/register/registration tracker/patient tracking/individual data	English only		6^11^^–^^13^

Abbreviations: K4Health, Knowledge for Health project; MAMA, Mobile Alliance for Maternal Action; PEPFAR, U.S. President's Emergency Plan for AIDS Relief; WHO, World Health Organization.

Using these search terms, we scanned the publication titles, abstracts, and executive summaries to determine their relevance to our research objectives. Only those resources that explicitly addressed issues of governance or stewardship were included. Despite requiring these terms in our search, we found that authors did not often directly address these issues. As a result, of the 404 resources we identified from the peer-reviewed literature, only 12 were deemed relevant. From the gray literature, only 6 resources were reviewed and included, based on quality, source, and relevance.

We read and analyzed the full text of the selected resources and abstracted information relevant to our stewardship themes—strategic direction, policies and procedures, roles and responsibilities, and health service delivery implications—and synthesized our results.

## RESULTS

We focused our review of the literature to identify lessons for digital health stewardship related to 4 broad thematic areas: strategic direction, policies and procedures, roles and responsibilities, and health service delivery implications. Other papers in this series will focus on financing, human resources, service provision, and service receipt.

Results of the literature review identified 4 broad areas—strategic direction, policies and procedures, roles and responsibilities, and health service delivery implications—where additional digital health stewardship research is needed.

### Strategic Direction

Although the overall available information was sparse, strategic direction was the theme most reflected in the publications reviewed. Importantly, although most of the work in this space has used the term ‘eHealth,’ the evolution of the conversation now appears to include ‘mHealth’ and, more broadly, ‘digital health,’ to which we believe that these findings also apply.

According to our research, strategic direction for digital health should address the following 6 topics^25^^,^^28^^–^^32^:

1. ***Responsibility for landscape scanning*** health sector needs as well as emerging innovations/solutions to identify priorities to pursue and potential stakeholders to involve
2. ***Establish consistency and comparability*** within the health sector and across sectors both for operations, such as definitions and standards, and benchmarks for privacy
3. ***Regulations and incentives to promote compliance*** with standards across stakeholders, which are reviewed and adapted to address emerging issues
4. ***Mechanisms for oversight and accountability*** of existing systems and introduction of new innovations, including concerns for equity
5. ***Structures to demand and support evidence-driven decisions*** for digital health, including introduction, expansion, and discontinuation of innovations and for how digital health outputs are used to inform others within and outside the public sector
6. ***Analysis of current and future availability of financing***, including approaches on how to address financing gaps

Further, the strategy should provide guidance on other themes, including which aspects of strategy implementation will be the government's responsibility as a public good, such as ICT infrastructure^16^ or data availability.

To establish an effective strategy, efforts must engage all stakeholders—including multiple government sectors, civil society, and private-sector actors—and target their needs and incentives in order to be successful.^25^ Private-sector actors for digital health present a unique case that is unlike most other health systems and service delivery areas. Although all actors have competing agendas and incentives, private-sector actors in the digital space, such as mobile network operators and technology developers, bring different objectives, expectations, and dynamics into the process, including profit orientation and shorter timeframes. MOHs in LMICs unfamiliar with these differences may consequently have less experience tailoring engagement and negotiation approaches that still ensure that all actors are held accountable.^33^ Data from the Global Observatory for eHealth suggests that public–private partnerships for eHealth are better leveraged by governments, which have more capacity and experience and, relatedly, are less affected by private-sector pushback on legislation that constrains their activities.^23^ Lang suggests that governments that develop eHealth legislation were able to do so by building on earlier experience, capacity, and resources for general governance and legislating.^23^

In terms of approaches, evidence from Europe indicates that certain settings are more successful in achieving effective strategies, such as in top-down health systems that are more directive and in countries that engage in policy dialogues.^20^ In the last decade, multiple LMICs, including the Philippines, Mozambique, Nigeria, Rwanda, and Tanzania,^34^ have started to create national approaches to eHealth. Despite these steps forward, the actual implementation of these strategies has been more complicated and time consuming than the countries expected.

The Broadband Commission for Sustainable Development has categorized 3 potential governance mechanisms that are likely to be effective: through the MOH, through a government-wide digital agency mechanism where the MOH is responsible for health issues, or through a dedicated third-party digital health agency with its own resources.^5^ Examples of institutions established in high-income countries can provide useful illustrations (Table 2). Institutional arrangements for eHealth are common in LMICs; they include (1) broadening the mandate of existing MOH units to include eHealth; (2) establishing technical working groups that include the public sector, external development partners, civil society, and the private sector; and (3) tasking parastatal institutions like national statistics offices with providing guidance.

**TABLE 2.** Examples of Digital Health Institutions

Example	Institutional Features	Mandate and Focus Areas
Canada Health Infoway (founded 2000)^16^	Independent, not-for-profit organization governed by 14 deputy ministers of health representing provincial authorities	Establishing strategic direction for electronic health records in Canada in collaboration with local authorities. Areas of focus include interoperability; information systems for registries, drugs, and labs; and innovation/adoption.
MedCom (established in 1994)^16^	Danish coordinating agency for health care IT. Danish central government contributes to health care IT through its National Board of Health.	Establishes standards for electronic data exchange.
eHealth Governance Initiative (established formally in 2011)^17^^,^^18^	Created with agreement of member states, and reports to high-level councils of the European Union	Created to promote eHealth services, establish strategies around eHealth, and help foster cooperation at the political, strategic, and operational levels. Four key areas of focus are identification and authentication of eHealth users, trust and acceptance of eHealth systems, legal issues (such as differing security and privacy requirements), and technical challenges.

Abbreviation: IT, information technology.

Three potential governance mechanisms may be effective: broadening the mandate of existing MOH units, establishing multi-stakeholder technical working groups, and tasking other government institutions, such as national statistics offices, with providing guidance.

These examples, both implemented and theoretical, reflect a range of potential institutional arrangements (e.g., parastatal, coordinating agency), with different characteristics (e.g., include multiple levels of government or several countries), and varying institutional objectives (e.g., contribute to national policy or regional cooperation). One aspect that several examples^17^^,^^19^^,^^21^ have in common is that strong political will from government actors was present at the outset, suggesting that this type of support, preferably broad-based, may be necessary for success. This level of government support is typified by the presence of digital health working groups, steering committees, or departments with clear mandates and defined linkages to health programs. Examples for these structures and positions can be found in national eHealth policies of Rwanda^35^ and Tanzania,^36^ which establish eHealth steering committees, propose the development of an eHealth department, and formalize the relationship between these groups and the MOH IT departments.

It is worth noting that whatever institutional structure is developed, significant flexibility in its outputs is crucial. Guidance generated can be practical (e.g., review process guidelines,^22^ ICT infrastructures for cloud computing^27^) as well as vision-oriented (e.g., member state cooperation^21^) as long as it serves the overall objective of providing direction to the sector.

### Policies and Procedures

Unlike strategic direction, the guidance on policies and procedures is more limited, and can be broken down into 2 mechanisms intended to carry out a strategic vision: high-level policies and implementation-oriented procedures. High-level policies are those that provide overarching guidance to the introduction, institutionalization, and eventual cessation of digital health innovations. Examples include:

- The 2004 eHealth Action Plan directs the European Commission to monitor the eHealth innovations landscape and promote best practices in sharing^20^
- The 2011 *mHealth: New Horizons for Health Through Mobile Technologies* document, as part of the WHO's Global Observatory for eHealth Series, identifies evaluation as a widespread barrier to adopting mHealth policies^31^
- The 2012 WHO and the International Telecommunication Union (ITU) *National eHealth Strategy Toolkit* provides guidance for developing national strategies on eHealth^32^
- The 2015 *Roadmap for Health Measurement and Accountability* provides standards for privacy and security^28^
- Additional publications from 2014 and 2015 examine country^27^ and regional^19^ efforts related to interoperability

Implementation-oriented procedures are routine processes necessary for assessing new technologies and innovations and for regularly assessing the health system for potential innovation opportunities. Ideally, these processes become institutionalized in the MOH so that they become standard practice by, for example:

- Harmonizing eHealth with existing laws, including privacy laws (e.g., patient data protection), liability for goods and services (e.g., cross-border telehealth), and trade and competition (e.g., online purchase of medicines)^19^
- Regular assessments of policy implications (e.g., cost-benefit analyses or regulatory impact assessments)^20^
- Design and implementation of governance that addresses consistency, testing, and introduction of new innovations^22^

A key aspect of stewardship policies and procedures is that it requires implementation to occur according to national and local norms and requirements. Sacks et al. describe the results of unclear stewardship of data management and access to sensitive health information related to the implementation of a digital contact-tracing program during the Ebola outbreak in Guinea.^37^ The implementers of the digital tool offered cloud-based data storage based on HIPAA (Health Insurance Portability and Accountability Act) privacy and ethical requirements from the United States, with the hope that these standards would be adequate. Although this approach was used at the beginning of the project, the National Ebola Coordinating Unit insisted on using local storage facilities for the data. This resulted in data being stored in 2 locations and a time lag that, for a period, made the final decisions of data ownership unclear, which also led to potential access and security complications. Had the local requirements been codified and understood at the national level from the beginning, the situation may have been avoided.^37^

A key aspect of stewardship policies and procedures is that it requires implementation to occur according to national and local norms and requirements.

### Roles and Responsibilities

The literature search revealed a lack of detailed information regarding the specific and operational staff roles and responsibilities required for a national ecosystem for digital health: some research focused specifically on the staff needed to use or manage end-stage digital tools,^22^ while others actively included developers, supervisors, trainers, and other implementation staff.^32^ Most recommendations are either broadly available as generic recommendations from global organizations^7^^,^^28^^,^^38^ or as specific project design recommendations from NGOs or implementers.^39^ The categories mentioned in the WHO and ITU *National eHealth Strategy Toolkit*^32^ are as comprehensive as any we identified, and include these 9 areas of responsibility:

- Program management
- Stakeholder engagement
- Strategic architecture
- Clinical safety
- Management and operation
- Monitoring and evaluation
- Policy oversight
- Health workforce
- Health ICT workforce

The combined recommendations for these 9 areas of responsibility total 1.5 pages of text, and concludes by stating that responsibilities exist at multiple levels of the health system and roles should be defined during implementation as a way of identifying “the preferred leadership and governance model, including defining the relationship to existing bodies at national, state, and local levels.”^32^

Country experience in Liberia suggested that clearly defining the role of existing project field staff to include routine data collection successfully reduced costs, enhanced supervision and ownership, and helped streamline the digital intervention to a manageable level of extra responsibilities.^40^ While context-specific models are important, a number of critical stewardship roles can be standardized across settings, as suggested by the Liberia example, such as responsibility for oversight of compliance with standards and laws or fundraising for system-wide improvements, which are often out of the purview of those actively using a device.^40^

While context-specific models are important, many critical stewardship roles can be standardized across settings.

### Health Service Delivery Implications

Digital health approaches offer the promise to address longstanding challenges with regard to equity, coverage, quality of care, and patient/consumer rights; however, very few publications addressed health service delivery implications.

Canada's Health Infoway was established in 2001 with the overarching goal of improving service delivery by reducing waiting time, improving patient safety in the community and institutions, improving quality of care, and improving efficiency and value for money.^17^ Hovenga indicates that health information governance, not just information, is needed in order to achieve improved health, responsiveness, social and financial risk protection, and efficiency.^25^ However, neither of these resources specifies what specific steps must be taken to achieve their objectives. This includes the key issue that the financial arrangements needed to sustain health IT implementation must address the rewards and incentives for digital health services improvement, which requires common ground across organizations and care providers to increase the quality of overall service delivery value system.^16^

Marshall, Lewis, and Whitaker describe a decision-making tool for allocating resources that could serve as a model for selecting digital health innovations for scale-up.^29^ The framework's criteria represent aspects of health service delivery that MOHs should be concerned with, including (1) physical access to technology; (2) appropriateness of technology; (3) affordability and use of technology; (4) human capacity and training; (5) locally relevant content, applications, and services; (6) integration into daily routines; (7) sociocultural factors; (8) trust in technology; (9) local economic environment; (10) macroeconomic environment; and (11) legal and regulatory frameworks. Another issue to resolve is related to citizens' rights to access health data and what entities (e.g., governments, external partners) are responsible for promoting the health data, especially when data are collected unidirectionally through mHealth activities without obvious benefit to individuals or communities.^30^

## DISCUSSION

In May of 2018, the World Health Assembly adopted a digital health resolution that calls on countries to assess and optimize their digital health offerings and “to identify areas where […] guidance and technical assistance and advice on digital health would be beneficial.”^2^ Although some of the topics mentioned included partial aspects of stewardship, stewardship and governance were never explicitly or formally described. The exclusion of stewardship in this key document corresponds with the results of our literature review, which shows that evidence-informed recommendations to facilitate national stewardship of digital health ecosystems are lacking. Guidance to facilitate strategic direction is often derived from generic stewardship recommendations or is limited to a discussion of eHealth that does not consider mHealth or the future direction of digital health, which will bundle a variety of technologies into a comprehensive ecosystem. Although recommendations related to stewardship have been around globally for years, practical ideas on institutionalizing robust digital health stewardship in LMICs have not made sufficient gains, as evidenced by similarly framed eHealth strategies developed years apart in Rwanda^35^ and Tanzania.^36^

The formation of potential digital health ecosystems has been primarily driven by a variety of actors outside of national health systems. Without formalized strategic direction, these organic fledgling ecosystems are unlikely to develop into efficient environments that will fully capitalize on the promise of digital innovations. As a result, governments will continue to face challenges of inefficiencies, competing systems, uncoordinated efforts, and unnecessary diversion of resources. A potential approach for addressing these challenges is to evaluate the successes and failures of LMICs that have attempted to develop national eHealth strategies and use those findings to support the funding and coordination of national (or regional) level multi-stakeholder workshops or policy dialogues to develop and/or update digital health strategies. At the same time, the best mechanisms through which to engage civil society and citizens at large remains elusive.

Without an adequately structured digital health ecosystem, governments will continue to face inefficiencies, competing systems, uncoordinated efforts, and unnecessary diversion of resources.

Private-sector engagement in digital health has the potential for both great opportunity and great risk. For instance, private-sector involvement from banking institutions or mobile network operators can suggest creative financing approaches that are less familiar or seem more risky to governments, such as mobile-based health savings schemes. Regardless of what options are considered, sustainability must remain at the forefront of any discussion. Another consideration is that countries with weaker governance or limited governing experience will likely not fare as well under public–private partnership arrangements; in those cases, external actors should be cautious of promoting these indiscriminately in LMICs.

With regards to policies and procedures, WHO observed in 2011 that policymaking was not keeping up with technological advances or the public's interest, especially around mHealth,^31^ and not much has changed since then. Cross-sectoral policy coordination, particularly with regards to the complex realities of technology, means that national policies often lag far behind digital health implementation. This not only results in challenges for digital health adoption but also opens up very real possibilities for misuse of health data, unverified health recommendations, and reduced adherence to clinical guidelines. Leaving this area of stewardship in the hands of outsiders has not resulted in many successes. This could be attributed in part to LMIC legislative and legal communities that have been largely uninvolved in the development of digital health policies to date. The development of a standard set of recommendations on a variety of policy topics, learning from but not copying the health regulations of wealthy countries, would ease the path for national discussions.

When national policies lag behind digital health implementation, these gaps enable misuse of health data, unverified health recommendations, and reduced adherence to clinical guidelines.

The roles and responsibilities for digital health stewardship is an area that could still be significantly improved. Many overarching models for eHealth strategies have been proposed, yet useful details of specific positions and responsibilities needed for a sustainable national ecosystem are largely absent. The organic process of adopting digital health into routine work procedures is not only slow but inadequate—many of the people in decision-making roles lack the training necessary to identify and hire for the skill sets needed to ensure proper long-term stewardship of a national digital health environment. A well-functioning digital health ecosystem in most countries will likely require such changes as the creation of new crosscutting units, new educational and training opportunities, a transformation of decision-making authority, revised reporting structures, and the delineation of legal authorities for data management and governance. Most donor-led health projects do not have the mandate or funding to focus on system-wide transformations, and most countries lack the incentives or resources to do so themselves. Funding for the development of a set of generic digital health staffing and responsibility recommendations would be a useful starting point. These recommendations could then be adapted for specific countries during or following the strategic and policy activities recommended above.

One outstanding issue has been how to manage the roles of external partners, including donors and NGO implementers, under new stewardship structures. The 2012 WHO and ITU *National eHealth Strategy Toolkit*^32^ includes extensive guidance relevant to digital health stewardship. The focus of the guidance is on developing an eHealth vision, or strategic direction, and an overarching action plan. The toolkit does highlight, however, that the successful application of a vision, strategy, or action plan requires experienced individuals to guide the core processes and engage with broader stakeholder groups, which echoes this finding of our review: local capacity and experience must be built and supported to ensure a long-lasting stewardship role.

Government actors must take the lead in any digital health stewardship initiative in order to reinforce country ownership, but to do this most LMICs will need capacity building. External partners have significant contributions to make not only in capacity building but also in supporting alternative approaches to stewardship. However, they must think beyond specific digital health applications and technologies and consider larger structural governance issues that reflect broader health systems issues that may need to be addressed. A regional stewardship initiative could be useful both from a country and donor perspective, as it would provide an opportunity for knowledge and practice sharing, increase the potential of scaling certain innovations, become the platform for cross-national interoperability, and address power imbalances between national governments and external actors. The Digital Regional East African Community Health Initiative recently launched a road map that includes a framework for unifying digital health approaches intended to support the cross-national environment necessary for digital health technologies to achieve sustainability and scalability.^41^ Although it is in its early stages, this type of effort underscores the potential of regional approaches in catalyzing gains in digital health.

Governments must lead digital health stewardship initiatives in order to reinforce country ownership, but to do so most countries require focused capacity building efforts.

### Limitations

While we gathered what we believe is a comprehensive picture of the literature, our review is not without limitations. Our review was a rapid purposive review and could have benefitted from a more systematic review, particularly of the gray literature. Additionally, we only included papers available in English or Spanish; there may be valuable publications available in other languages. Although we kept our country scope generally broad in order to better capture the state of the field, we recognize that not all high-income country approaches are easily transferable to LMICs. Further theoretical and practical work is needed to translate and adapt appropriate approaches to different contexts.

## CONCLUSIONS

While a lot has been written about the potential of digital health to address longstanding challenges with health service delivery, better guidance is needed to help countries use digital health in transformative, rather than replicative, ways. Despite the importance of national stewardship for achieving the global health community's goals for digital health—outlined in *The Roadmap for Health Measurement and Accountability*,^28^ the Principles for Digital Development's principles,^38^ and implied in the goal of universal health coverage within the third Sustainable Development Goal^42^—significant work remains to standardize and operationalize recommendations. Formal focus and attention on the areas of stewardship and governance still needs considerable attention, even by the most current and high-profile actors in this space. While a fair amount of relevant literature in this space has been published, most of it has not been peer reviewed, does not reflect LMIC experiences, and—more importantly—has not been replicated. Most of the guidance available is aspirational in tone, informed by stand-alone projects, or highlighting the difficulty of the task at hand. Yet the promise of digital health is undeniable. To help make aspirations a reality, specific efforts should be undertaken to improve national stewardship that reflects a country-owned vision of how digital health can catalyze health system achievements.

## Notes

**Peer Reviewed**

**Funding:** This work was made possible by a grant from the Aetna Foundation, with support from the World Health Organization and The Johns Hopkins University Global mHealth Initiative.
